# Dysregulated iron metabolism associates with neutrophilic airway inflammation in COPD

**DOI:** 10.1042/CS20257442

**Published:** 2026-02-17

**Authors:** James Baker, Andrew Higham, Christopher McCrae, Mohammadali Yavari Ramsheh, Christopher Brightling, Simon Lea, Dave Singh

**Affiliations:** 1 Division of Immunology, Immunity to Infection and Respiratory Medicine, School of Biological Sciences, Faculty of Biology, Medicine and Health, Manchester Academic Health Science Centre, The University of Manchester and Manchester University NHS Foundation Trust, Manchester, U.K.; 2 Translational Science and Experimental Medicine, Early Respiratory & Immunology, BioPharmaceuticals R&D, AstraZeneca, Gaithersburg, MD, U.S.A.; 3 Imperial Centre for Translational and Experimental Medicine (ICTEM), Department of Surgery & Cancer, Faculty of Medicine, Imperial College London, London, U.K.; 4 Department of Respiratory Sciences, University of Leicester, Leicester, U.K.; 5 Medicines Evaluation Unit, The Langley Building, Southmoor Road, Manchester University NHS Foundation Trust, Manchester, U.K.

**Keywords:** Chronic obstructive pulmonary disease, Endothelial dysfunction, heme, Iron, Neutrophils

## Abstract

Pulmonary iron levels are increased in chronic obstructive pulmonary disease (COPD), possibly due to increased red blood cell leakage from the microvasculature. Neutrophils cause endothelial cell damage which may cause vascular dysfunction and iron dysregulation in COPD. We investigate the relationships between neutrophilic inflammation, iron metabolism and vascular dysfunction in COPD. Using gene and protein analysis, associations between neutrophilic inflammation, iron dysregulation and vascular dysfunction were investigated in two COPD bronchoscopy cohorts: EvA (*n*=51) and Manchester (*n*=33). Patients were sub-grouped based on bronchoalveolar lavage (BAL) neutrophil percentage (neutrophil^high^≥3% and neutrophil^low^<3%). Heme was measured in BAL by LC-MS. BAL cell gene expression of neutrophilic inflammation markers such as C-X-C Motif Chemokine Ligand 8 (CXCL8) and interleukin 6 receptor (IL6R) were significantly increased in neutrophil^high^ compared with neutrophil^low^ patients in both cohorts; fold change (FC) differences 1.06–17. We found increased markers of iron and iron trafficking including lactoferrin (LTF), lipocalin-2 (LCN2) and myoglobin (MB) in neutrophil^high^ patients in both cohorts. BAL cell gene expression and BAL fluid protein levels of the vascular dysfunction marker, vascular endothelial growth factor (VEGF), were significantly higher in neutrophil^high^ compared with neutrophil^low^ patients. Fibrinogen and heme were significantly increased in neutrophil^high^ BAL fluid. *In vitro* experiments revealed that blood neutrophils had significantly increased expression of LTF and VEGFA following LPS-stimulation and heme induces endothelial dysfunction. COPD patients with distal lung neutrophilic inflammation have dysregulated iron metabolism which may be a consequence of increased vascular leakage into the airways.

## Introduction

Chronic obstructive pulmonary disease (COPD) is characterised by airflow limitation caused by the inhalation of noxious particles, such as cigarette smoke [1]. Airway inflammation and tissue remodelling are hallmarks of COPD, resulting in small airway disease and emphysema [2]. Neutrophil numbers are increased in the lungs of COPD patients; these cells form a first line of defence against invading pathogens [3] using various anti-microbial strategies including phagocytosis, superoxide release, degranulation, secretion of iron binding proteins and neutrophil extracellular trap formation [3–5]. However, prolonged neutrophil activation may also cause tissue damage, including to the endothelial barrier, due to increased protease release [6,7].

Under homeostatic conditions iron levels in the lung are tightly regulated by extracellular iron binding proteins, including lactoferrin (LTF), haptoglobin (HP) and lipocalin-2 (LCN2) that bind free iron in a non-redox active form. This allows iron uptake by import proteins such as transferrin receptor (TFRC), cluster of differentiation 163 (CD163) and low-density lipoprotein receptor-related protein 1 (LRP1) [8]. Pulmonary iron levels are increased in COPD bronchoalveolar lavage (BAL) fluid and alveolar macrophages [9–11]. Increased lung iron levels contribute to oxidative stress in COPD through the generation of free radicals by the Fenton reaction. Despite increased oxidative stress in COPD, expression of anti-oxidative Nrf2 pathway enzymes are reduced compared with controls [12], suggesting a dysfunctional anti-oxidative stress response in COPD patients.

Endothelial damage and vascular leakage of red blood cells into the alveolar space have been proposed as a potential source of increased alveolar iron levels in COPD [11,13]. In support of this, we have shown that red blood cell coverage in the alveolar space is increased in COPD [11]. Activated neutrophils contribute to endothelial damage in COPD, thereby potentially contributing to red cell leakage [6]. Patients with α-1 antitrypsin (AAT) deficiency have dramatically increased BAL iron and heme levels, highlighting the role of neutrophil derived proteases in the leakage of heme and iron into the alveolar space [14].

We hypothesised that increased lung neutrophil numbers in COPD are associated with a distinct profile of inflammation, iron dysregulation and vascular dysfunction. We conducted gene and protein analysis to investigate the relationships between neutrophilic inflammation, iron metabolism and vascular dysfunction in BAL samples from two COPD cohorts. We also assessed the interactions between neutrophil activation, iron metabolism and endothelial dysfunction *in vitro*.

## Methods

### Study subjects

COPD patients aged >40 with a smoking history of >10 pack-years, a post-bronchodilator forced expiratory volume in 1 s (FEV_1_) and forced vital capacity (FVC) ratio <0.7 were recruited. Patients receiving oral corticosteroids or antibiotics within six weeks of the study were excluded.

Samples obtained from two previously described COPD cohorts were used: the Emphysema versus Airways Disease (EvA) study [15], composed of ex-smokers only and a Manchester cohort [16]. Only COPD patients with BAL differential cell counts were included; Eva cohort *n*=51 and Manchester cohort *n*=33.

This study was conducted in accordance with the Declaration of Helsinki 1975. Sample collection was approved by the local research ethics committees, and written informed consent was obtained from study participants.

### Bronchoscopy samples

Bronchoscopy was performed after sedation and BAL was collected. The aspirated fluid was filtered and centrifuged before the BAL fluid was removed and the cell pellet was stored in RNA protect (EVA cohort) or in RLT buffer plus β-mercaptoethanol (Manchester cohort) prior to RNA extraction.

Cytospins were prepared before being air-dried, fixed in methanol, and stained with RapiDiff (Triangle, Skelmersdale, U.K.) for differential cell counting. We separated COPD patients into two groups based upon BAL neutrophil percentages using the differential cell count according to ATS guidelines: neutrophil^low^ <3% and neutrophil^high^ ≥3% [17].

### Gene expression analysis

We investigated a discrete set of genes and proteins in a hypothesis driven approach; genes associated with neutrophilic inflammation in previous COPD studies were selected for analysis, as well as proteases and anti-proteases [18–20], iron metabolism genes [21] and vascular dysfunction markers identified from previous publications [6,22] (full gene names listed in Supplementary Table S1).

### Protein analysis

Protein analysis was available for the Manchester cohort only. BAL fluid was assessed for CXCL5, CXCL8, IL-1α, IL-1β, IL-6, IL-6r, SLPI, AAT, A2M, MMP-9, TIMP-1, LTF, HP, MB, B2M, FTH1, Fibrinogen, VEGF, and ANGPT2 by multiplex assay (Myriad RBM). Protein levels were normalised to patient-specific BAL urea concentrations, measured by colorimetric assay (Biovision Inc). Data were not available or below the limit of detection for other targets examined by gene expression analysis.

### Heme and iron measurement

BAL fluid heme was measured by Liquid chromatography-mass spectrometry (LC-MS). Iron was measured by Inductively Coupled Plasma Mass Spectrometry (ICP-MS) (Agilent 7700x ICP-MS). Full details of heme and iron measurement are in the online supplement.

### Neutrophil activation

Neutrophils were isolated using EasySep Neutrophil Isolation Kit CAT: 17957 (StemCell). Neutrophils were then treated with LPS (0.1 μg/ml) for 3 h. Gene expression was assessed by qPCR with full details in the online supplement.

### Endothelial barrier function

Endothelial cells (HULEC-5a) (ATCC) were treated with heme (0–100 μM) for 24 h. Cell viability was measured by 3-(4,5-dimethylthiazol-2-yl)-2,5-diphenyltetrazolium bromide (MTT) assay; Trans-endothelial resistance (TEER) was measured by EVOM2 voltmeter (World Precision Instruments); CXCL8 and IL6 were measured by ELISA (R&D systems). Further details are in the online supplement.

### Statistical analysis

Statistical analyses were performed using GraphPad InStat software (GraphPad Software Inc). Data distributions were determined by the D’Agostino and Pearson normality test. Further details are in the online supplement.

## Results

### Study subjects

The EVA cohort included 22 neutrophil^high^ and 29 neutrophil^low^ patients and the Manchester cohort included 16 neutrophil^high^ and 17 neutrophil^low^ patients, defined using BAL cell counts.

The clinical characteristics of the study populations are presented in Table 1, with the neutrophil^high^ and neutrophil^low^ COPD patients well matched in both cohorts. The Eva cohort comprised of a mix of GOLD I and GOLD II patients whereas the Manchester cohort was mostly GOLD II patients. The Eva cohort contained only ex-smokers while the Manchester cohort included both current and ex-smokers. As expected, there were significant differences in BAL neutrophil and macrophage percentages (*P*<0.0001) between the neutrophil^high^ and neutrophil^low^ groups. In the EVA cohort, sputum neutrophil percentage plus blood neutrophil and lymphocyte counts were significantly higher in the neutrophil^high^ compared with neutrophil^low^ patients (*P*<0.05).

**Table 1:** Clinical characteristics and cell counts of the EVA and Manchester study population

	EVA cohort			Manchester cohort		
Clinical characteristics	<3%	>3%	*P*-value	<3%	>3%	*P*-value
*n*	29	22		17	16	
Age (years)	65 ± 6	64 ± 7	0.5	62 ± 6	62 ± 4	0.8
Gender: male *N* (%) ‡	20 (69)	12 (55)	0.3	10 (59)	11 (62)	0.9
Current Smoker *N* (%) ‡	0	0	N/A	10 (59)	7 (44)	0.5
Pack years	44 ± 16	36 ± 18	0.1	43 ± 17	37 ± 13	0.3
BMI (kg/m^2^)	29 ± 6	28 ± 5	0.5	25 ± 5	26 ± 4	0.7
Exacerbation rate (in previous year)	NR	NR	N/A	0.6 ± 0.9	1.0 ± 1.2	0.3
mMRC	1.0 ± 0.9	1.0 ± 0.6	1.0	1.2 ± 0.7	1.9 ± 1.3	0.07
CAT				16 ± 6	20 ± 10	0.2
SGRQ				45 ± 19	36 ± 14	0.2
FEV_1_ (L)	2.2 ± 0.5	2.1 ± 0.6	0.6	1.8 ± 0.3	1.9 ± 0.4	0.5
FEV_1_% predicted	81 ± 15	79 ± 20	0.7	65 ± 10	63 ± 11	0.6
FEV_1_/FVC ratio	61 ± 7	60 ± 8	0.7	53 ± 8	52 ± 8	0.7
ICS users (%) ‡	38	50	0.4	65	69	0.8
LAMA users (%) ‡	41	32	0.5	53	69	0.4
LABA users (%) ‡	48	55	0.7	56	63	0.7
No maintenance inhaled medication (%) ‡	34	23	0.4	18	0	0.1
GOLD I (%) ‡	59	45	0.4	6	0	0.2
GOLD II (%) ‡	41	50	0.4	94	88	0.2
GOLD III (%) ‡	0	5	0.4	0	12	0.2
**BAL characteristics**						
Neutrophil (%) †	1 (0–3)	9 (3–97)	<0.0001	1 (0.3–3)	11 (5–76)	<0.0001
Macrophage (%) †	92 (60–97)	74 (2–88)	<0.0001	96 (55–99)	73 (4–91)	<0.0001
Eosinophil (%) †	0.3 (0–2.3)	0.3 (0–4.3)	0.5	0.5 (0–1.8)	0.8 (0–8.0)	0.1
Lymphocyte (%) †	7 (2–40)	8 (0–33)	1	1 (0–43)	2 (0–17)	0.6
**Sputum characteristics**						
Neutrophil (%) †	58.4 (20–82)	77.1 (49–98)	0.02	75 (17–94)	67 (15–91)	0.9
Eosinophil (%) †	3.1 (0–11)	2.4 (0–10)	0.5	1.3 (0–29)	3.5 (0–70)	0.2
**Blood characteristics**						
Neutrophil (×10^9^/l) †	4.0 ± 1.2	4.7 ± 1.2	0.03	4.3 ± 1.4	4.1 ± 1.3	0.7
Eosinophil (×10^9^/l) †	0.2 ± 0.1	0.2 ± 0.1	0.8	0.2 ± 0.2	0.3 ± 0.2	0.2
Lymphocyte (×10^9^/l) †	1.9 ± 0.7	2.4 ± 0.8	0.03	2.0 ± 0.6	2.1 ± 0.7	0.9
Haemoglobin (gm/dL)	14.37 ± 1.1	14.30 ± 1.3	0.84	14.9 ± 1.9	14.3 ± 1.1	0.17

Data are presented as mean ± standard deviations or median (range). †: Mann–Whitney *U*-test. ‡: Fisher’s exact test and where not indicated an unpaired *t*-test was carried out. BMI, body mass index; mMRC, modified medical research council dyspnea scale; FEV1, forced expiratory volume in one second; FVC, forced vital capacity; ICS, inhaled corticosteroid; LAMA, long-acting muscarinic antagonist; LABA, long-acting β-agonist; NR, not reported (NR). Lung function data are post-bronchodilator.

### Inflammation markers

#### Gene expression

**Figure 1:**
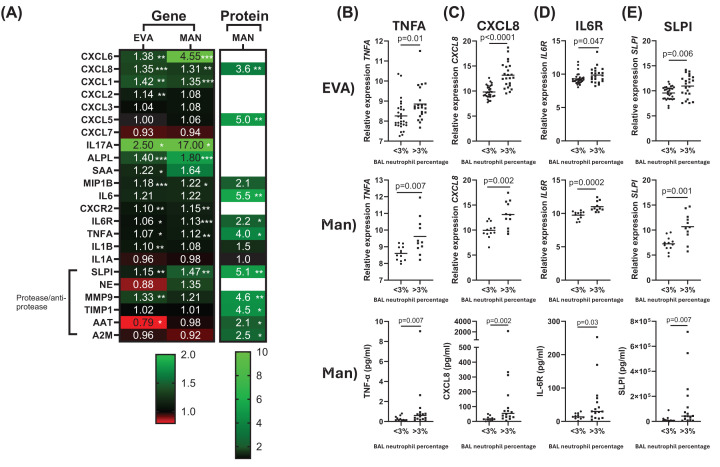
Neutrophilic inflammation marker gene expression and protein levels in neutrophil^high^ and neutrophil^low^ patients (**A**) Gene expression was assessed in BAL cells (primarily macrophages, neutrophils, eosinophils and lymphocytes). Protein levels were assessed in BAL fluid. Fold change difference of neutrophil^high^ gene expression and protein levels compared with neutrophil^low^ is presented as a heatmap. Comparisons between groups were made by an unpaired *t*-test for gene expression and protein levels. *P<0.05, **P<0.01 and ***P<0.001. Selected genes/proteins presented graphically: (**B**) TNFA, (**C**) CXCL8, (**D**) IL6R and (**E**) SLPI. Data are presented as individual values where the black horizontal line presents the mean. CXC Motif Chemokine Ligand 1 (CXCL1), CXC Motif Chemokine Ligand 2 (CXCL2), CXC Motif Chemokine Ligand 3 (CXCL3), CXC Motif Chemokine Ligand 5 (CXCL5), CXC Motif Chemokine Ligand 6 (CXCL7), CXC Motif Chemokine Ligand 7 (CXCL7), CXC Motif Chemokine Ligand 8 (CXCL8), CXC motif chemokine receptor 2 (CXCR2), Alkaline phosphatase (ALPL), Interleukin-1 alpha (IL1A), Interleukin-1 beta (IL1B), Tumor necrosis factor alpha (TNFA), Interleukin 6 (IL6), Interleukin-6 receptor (IL6R), Macrophage inflammatory protein-1 (MIP1B), Interleukin-17A (IL17A), Serum amyloid A (SAA), Neutrophil elastase (NE), Alpha-1 antitrypsin (AAT), Secretory Leukocyte Peptidase Inhibitor (SLPI), Matrix metalloproteinase 9 (MMP9), TIMP Metallopeptidase Inhibitor 1 (TIMP1) and Alpha-2-Macroglobulin (A2M).

The threshold of 3% BAL neutrophils identified the following inflammatory genes with increased expression (*P*<0.05) in neutrophil^high^ compared with neutrophil^low^ patients in both the EVA and Manchester cohorts with fold change (FC) differences ranging from 1.06 to 17.00; *CXCL6, CXCL8*, *CXCL1*, *IL17A*, *ALPL*, *CXCR2*, *IL6R*, *MIP1B* and *TNFA* (Figure 1 and Supplementary Table S2). Additionally, the anti-protease *SLPI* was significantly increased (*P*<0.001) in neutrophil^high^ compared with neutrophil^low^ patients in both cohorts (FC 1.15 and 1.47, respectively). *SAA, IL1B* and *MMP9* were significantly increased in the EVA cohort but the Manchester cohort results did not reach significance (*P*=0.05, *P*=0.08 and *P*=0.06, respectively). All other genes were not differentially expressed in either cohort.

#### Proteins

**Figure 2:**
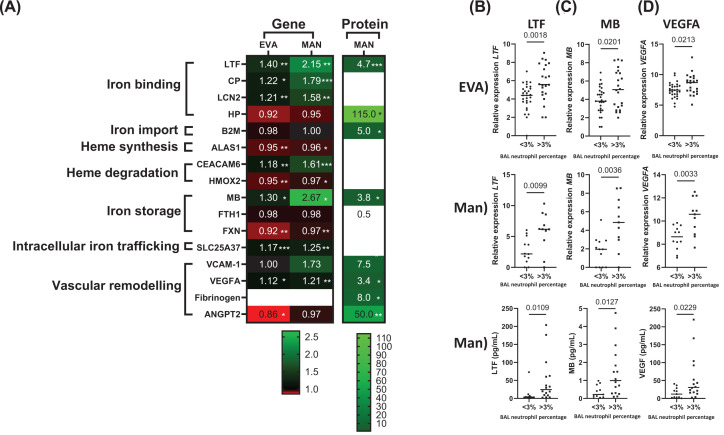
Iron metabolism marker gene expression and protein levels in neutrophil^high^ and neutrophil^low^ patients (**A**) Gene expression was assessed in BAL cells (primarily macrophages, neutrophils, eosinophils and lymphocytes). Protein levels were assessed in BAL fluid. Fold change difference of neutrophil^high^ gene expression and protein levels compared with neutrophil^low^ is presented as a heatmap. Comparisons between groups were made by an unpaired *t*-test for gene expression and protein levels. *P<0.05, **P<0.01 and ***P<0.001. Selected genes/proteins presented graphically: (**B**) LTF, (**C**) MB and (**D**) VEGFA. Data are presented as individual values where the black horizontal line presents the mean. Lactoferrin (LTF), Haptoglobin (HP), Ceruloplasmin (CP), Lipocalin-2 (LCN2), Delta-aminolevulinate synthase 1 (ALAS1), Heme Oxygenase 2 (HMOX2), Carcinoembryonic antigen-related cell adhesion molecule 6 (CEACAM6), Ferritin heavy chain (FTH1), Frataxin (FXN), Myoglobin (MB), Mitoferrin-1 (SLC25A37), Vascular cell adhesion protein 1 (VCAM-1), Vascular endothelial growth factor A (VEGFA) and Angiopoietin-2 (ANGPT2).

Protein analysis of BAL fluid in the Manchester cohort showed significantly increased levels of CXCL8, CXCL5, IL6, IL6R and TNFα in neutrophil^high^ patients (*P*<0.05, Figure 1 and Supplementary Table S2). For the proteases/anti-proteases, there were significant increases in SLPI, MMP9, TIMP1, AAT and A2M levels in neutrophil^high^ patients (*P*<0.05). Of these proteins, TNFα, CXCL8, IL6R and SLPI also had significantly higher gene expression in neutrophil^high^ patients in both cohorts (*P*<0.05); these individual gene and protein results are shown together in Figure 2B–E. TNFα, CXCL8, IL6R and SLPI protein levels were 4-, 3.6-, 2.2- and 7.7-fold greater, respectively, in neutrophil^high^ patients. The expression of these four genes were significantly correlated with BAL neutrophil %: (rho = 0.5–0.8, *P*<0.01 for all comparisons, Supplementary Figure S1) while at the protein level, only IL6R was significantly correlated with neutrophil % (rho = 0.48, *P*=0.001, Supplementary Figure S2).

### Iron metabolism and vascular remodelling

#### Gene expression

The expression of iron metabolism and vascular remodelling genes that were differentially expressed according to neutrophil counts are shown in Figure 2 and Supplementary Table S3. Additionally, all genes with protein data available are shown. Genes that were not differentially expressed in both studies are shown in Supplementary Table S4. Increased gene expression in neutrophil^high^ patients in both cohorts was observed for the iron binding genes LTF, CP and LCN2 (FCs ranging from 1.2 to 2.1; *P*<0.05), the iron trafficking protein SLC25A37 (FC 1.17 and 1.25; *P*<0.01), the negative regulator of heme-oxygenases CEACAM6 (FC 1.18 and 1.61; *P*<0.01) and the heme-containing protein MB (FC 1.3 and 2.67; *P*<0.05). Significant down-regulation in neutrophil^high^ patients in both cohorts was observed for the heme synthesis gene ALAS1, the heme metabolism gene HMOX2 (0.95–0.97; *P*<0.05) and the mitochondrial iron storage protein FXN, (FC 0.92 and 0.97; *P*<0.001) although the FC were small for these downregulated genes.

Expression of the vascular dysfunction marker VEGFA was significantly higher in neutrophil^high^ compared with neutrophil^low^ patients with a FC of 1.12 and 1.21 (*P*<0.05).

#### Protein

Amongst the genes with significantly increased expression in neutrophil^high^ compared with neutrophil^low^ patients in both cohorts, we also observed higher LTF, MB and VEGF protein levels in neutrophil^high^ patients with FC differences of 4.7, 3.8 and 3.4, respectively (*P*<0.05) (Figure 2 and Supplementary Table S3). VEGFA correlated with BAL neutrophil % (rho = 0.49, *P*=0.008, Supplementary Figure S3), while LTF did not reach significance (rho = 0.37, *P*=0.053) and MB showed no significant correlation.

HP, B2M, fibrinogen and ANGPT2 protein levels were significantly increased in neutrophil^high^ patients but there were no differences for gene expression (Figure 2 and Supplementary Table S3). VCAM-1 levels were increased in neutrophil^high^ patients but did not reach significance (*P*=0.05).

#### BAL iron and heme levels

**Figure 3:**
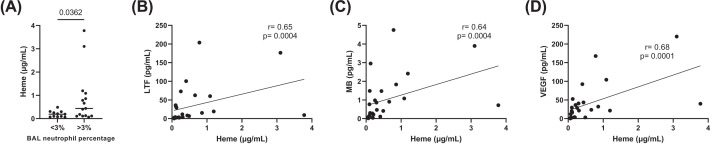
BAL heme and iron/endothelial protein levels levels in neutrophil^low^ and neutrophil^high^ COPD patients (**A**) BAL heme was measured by Liquid chromatography-mass spectrometry (LC-MS) in the Manchester cohort: neutrophil^low^*n*=11 and *n*=15 neutrophil^high^ COPD patients. Data are presented as individual values where the black horizontal line presents the mean. BAL heme was plotted against (**B**) LTF, (**C**) MB and (**D**) VEGF protein levels. Data are presented as individual values with linear regression plotted.

Heme levels in BAL fluid were significantly increased in neutrophil^high^ compared with neutrophil^low^ patients (0.44 vs 0.2 μg/ml, respectively, *P*=0.036; Figure 3). We evaluated the associations between heme and LTF, MB and VEGF protein levels (as these were increased at the gene and protein level in neutrophil^high^ patients); heme levels were significantly correlated with levels of LTF (rho = 0.65, *P*=0.0004), MB (rho = 0.64, *P*=0.0004) and VEGF (rho = 0.68, *P*=0.0001; Figure 3).

Iron levels were not significantly different in neutrophil^high^ patients compared with neutrophil^low^ (Supplementary Figure S4, *P*>0.05). LTF levels were significantly correlated with BAL iron levels (rho = 0.46, *P*=0.01) while no association was observed for MB or VEGF (Supplementary Figure S4).

#### Iron and vascular gene/protein expression in activated neutrophils

**Figure 4:**
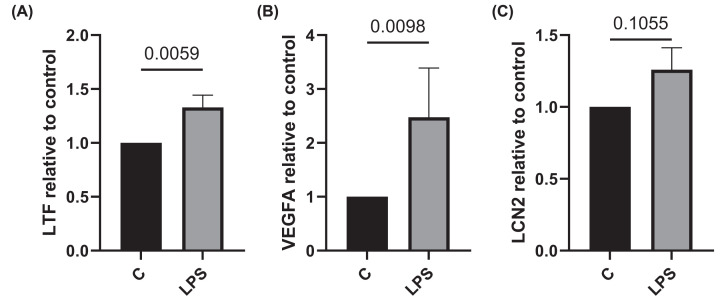
Iron and endothelial marker expression in activated neutrophils Blood neutrophils isolated from COPD patients (*n*=10) were treated with 0.1 μg/ml of LPS for 3 h. Gene expression of (**A**) LTF, (**B**) VEGFA and (**C**) LCN2 is shown relative to untreated controls. Comparisons between groups were made by a paired *t*-test.

Following observations of increased iron and vascular markers in neutrophil^high^ patients, we investigated the role of neutrophil activation in expression of LTF, MB, VEGF and LCN2. LTF and VEGFA expression were significantly increased in neutrophils activated by LPS (FC: 1.33 and 2.47, *P*=0.0059 and *P*=0.0098, respectively) (Figure 4). While LCN2 was numerically increased FC: 1.26, this did not reach significance (*P*=0.1). MB expression was not detectable (data not shown). LTF, LCN2 and VEGF protein secretion were numerically increased without reaching statistical significance increased following LPS exposure (Supplementary Figure S5).

#### Heme and endothelial function in vitro experiments

**Figure 5:**
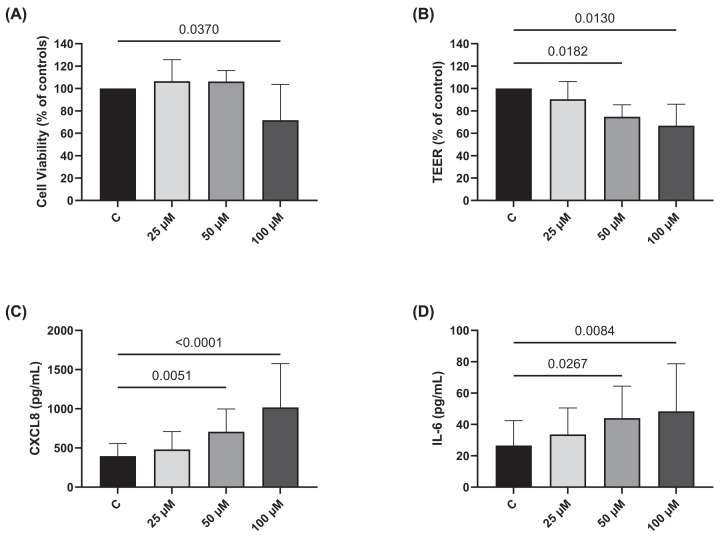
Endothelial barrier function and neutrophil chemokine/cytokine production in lung endothelial cells Lung endothelial cells (HULEC-5a) were treated with heme (0, 25, 50 and 100 μM) for 24 h. (**A**) Cell viability was assessed by MTT assay and is shown relative to untreated controls (*n*=11). (**B**) Barrier function was assessed by TEER and is expressed relative to untreated controls (*n*=8). (**C**) CXCL8 and (**D**) IL6 production were assessed by ELISA following heme treatment (*n*=7). Comparisons between groups were made by one-way ANOVA.

As heme was significantly increased in neutrophil^high^ patients we investigated the effect of heme on endothelial barrier function. Heme significantly reduced lung endothelial cell viability at 100 μM: (71.7%, *P*=0.037) with no effect at lower concentrations (25 and 50 μM) (Figure 5). TEER was reduced at all three concentrations (25 μM: 90%, 50 μM: 75% and 100 μM: 67%) and reached significance for 50 μM and 100 μM (*P*=0.018 and *P*=0.013, respectively). CXCL8 and IL6 production were significantly increased at 50 and 100 μM: *P*<0.05 (Figure 5).

## Discussion

Using samples from two independent cohorts, we report that greater distal airway neutrophilic inflammation in COPD patients is associated with dysregulated iron (heme) metabolism and evidence of endothelial dysfunction. Neutrophil^high^ COPD patients had higher levels of heme in BAL fluid and increased levels of biomarkers of iron binding and iron trafficking; overall, these findings indicate an environment with increased iron burden. The increased BAL fluid levels of fibrinogen and heme proteins, which are primarily blood-derived factors, implicates increased vascular leakage in neutrophil^high^ patients. Neutrophilic inflammation was also associated with increased protein levels of the vascular remodelling proteins VEGF and ANGPT2. These findings regarding vascular leakage and vascular remodelling highlight endothelial dysfunction as a potentially important pathophysiological process in COPD patients with greater neutrophilic airway inflammation.

Using a 3% BAL neutrophil threshold, we were able to identify a subgroup of COPD patients with a distinct profile of innate markers of inflammation. This neutrophilic endotype was investigated further for dysregulation of iron metabolism and vascular dysfunction. The novelty of this study is that we demonstrate that this inflammatory endotype was associated with vascular dysfunction that may be the cause for dysregulated iron metabolism. The use of two independent cohorts for gene expression plus protein analysis in one cohort decreases the chance that of false positive findings.

Increased vascular leakage into the distal airspaces has the potential to cause iron-induced toxicity and inflammation [23], and therefore a local response is required to counter the impact of increased iron. In support, we observed increased expression of the iron-binding molecules LTF, LCN2 and CP in neutrophil^high^ patients. Increased iron sequestration by iron binding proteins may explain why increased levels of heme but not total iron levels in BAL fluid were observed in neutrophil^high^ patients. To further understand these observations from clinical samples, we performed *in vitro* experiments with neutrophils and endothelial cells. Heme exposure reduced endothelial barrier function and caused cell death as shown previously [24]. We have shown a novel role of heme in causing the endothelial production of inflammatory mediators including the neutrophil chemoattractant CXCL8. These results demonstrate the potential for heme to (a) facilitate vascular leakage which may enable red cell influx into the lungs and hence iron overload and (b) promote neutrophilic inflammation. We also demonstrate that activated neutrophils show increased expression levels of genes related to iron metabolism. These observations from clinical cohorts and *in vitro* experiments show a potential positive feedback loop centred around the actions of heme which can cause endothelial leakage either directly or indirectly through the activation of neutrophils (discussed below); red blood cell leakage further increases heme levels thereby creating a positive feedback loop (summarised in Supplementary Figure S6) [11].

A strength of this study is the use of two independent cohorts to identify significantly dysregulated gene expression, with protein confirmation from one cohort available for the genes investigated. We had a pre-defined hypothesis that neutrophil^high^ patients have evidence of vascular leakage due to endothelial damage by neutrophils, resulting in dysregulated lung iron metabolism. This involved investigation of a discrete set of genes involved in innate immune signalling, iron metabolism and vascular endothelial function. While we did not correct for multiple testing within the gene sets, we reduced the chance of false positives by only identifying genes when positive in both cohorts. The protein analysis, discussed below, also decreased the chance of false positives.

The protein levels of fibrinogen, HP and ANGPT2, were increased in neutrophil^high^ patients despite no differences in gene expression. The explanation for these discordant observations is that these proteins are primarily produced outside the lungs; fibrinogen and HP are primarily expressed by hepatocytes and secreted into the circulation [25,26] whereas ANGPT2 is produced in large quantities by hepatocytes and endothelial cells. For these proteins it is likely that increased vascular leakage in neutrophil^high^ patients caused increased levels in BAL fluid, as neutrophil proteases degrade the endothelial barrier [6,7,27]. Indeed, VEGF and ANGPT2 are key drivers of angiogenesis and are increased in response to endothelial damage and vascular remodelling caused by neutrophils in COPD [6,22,28,29].

Lodge et al. showed that hypoxic neutrophils caused endothelial barrier damage in COPD [6]; importantly, endothelial damage required activation of neutrophils by hypoxia as neutrophils in a low activation state had no effect. We confirm previous observations of increased VEGF gene expression following activation of neutrophils *in vitro* and extend these findings demonstrating LTF gene expression is induced following neutrophil activation [30]. While LTF, LCN2 and VEGF protein secretion was increased following LPS stimulation, this did not reach significance. Protein levels may not have reflected the transcriptional changes induced by LPS at this time point. We did not observe a significant increase in expression LCN2 following LPS stimulation in normoxic conditions (21% O_2_) suggesting other stimuli such as hypoxia may be required as evidenced by Lodge et al*.* [6]. Furthermore, we observed increased protein levels of the anti-protease SLPI and protease MMP9 suggesting an environment with dysregulated protease activity that may contributing to vascular remodelling in the neutrophil^high^ group [31]. We propose that neutrophil^high^ patients may represent a distinct endotype in COPD characterised by neutrophil mediated damage of the endothelium and dysregulated iron metabolism. This patient group may be susceptible to greater tissue damage and therefore benefit from novel therapeutics targeting innate immune pathways including neutrophils.

MB is expressed in skeletal muscle, endothelial cells and smooth muscle cells primarily functioning as an oxygen transport protein [32]. MB gene expression and protein levels were higher in neutrophil^high^ patients. MB can scavenge ROS and induce endothelial cell apoptosis [33,34]. Further work is needed to establish the role of MB in COPD associated neutrophilic inflammation and endothelial function.

The BAL neutrophil^high^ group had significantly higher sputum neutrophil percentages than neutrophil^low^ for EVA but not the Manchester cohort. This may be due to the Manchester cohort containing a mix of current and ex-smokers; sputum neutrophil numbers are lower in COPD current smokers compared to COPD ex-smokers [35]. Sputum studies have implicated IL6, IL6R, IL1B and CXCL8 in IL-6 trans signalling (IL-6TS), which is strongly associated with increased neutrophil numbers [20]. Here, IL6R and CXCL8 were up-regulated in BAL neutrophil^high^ patients which is consistent with the IL-6TS results from sputum. Differences between sputum and BAL studies may reflect distinct patterns of inflammation in the upper versus lower airways, or methodological differences between assays [36].

IL17A was the most highly up-regulated gene in neutrophil^high^ patients in both cohorts (FC: 2.5 and 17.00), in agreement with studies demonstrating the association of IL17A and neutrophilic inflammation in COPD [37]. IL17A may also play a role in the COPD endothelial dysfunction as murine models demonstrate IL17As association with neutrophil recruitment, vascular leakage and vascular remodelling [38].

Gene expression of the mitochondrial iron storage molecule FXN was decreased in neutrophil^high^ patients. Decreased FXN has been associated with increased mitochondrial heme in a mouse model of COPD [39]. We speculate that increased mitochondrial heme/iron levels may occur in neutrophil^high^ patients as evidenced by reduced FXN. Increased mitochondrial iron is known to induce mitochondrial dysfunction to the point of mitophagy, a process of selective degradation of mitochondria which has been implicated in COPD pathogenesis [40].

Our study was cross-sectional which limits our ability to determine whether gene/protein signatures are stable over time or whether they are modulated by acute inflammatory events. A limitation of this study was that investigation of activation status of neutrophils in neutrophil^high^ compared with neutrophil^low^ patients was not possible due to the retrospective nature of this study. *In vitro* neutrophil experiments were conducted in an independent cohort of patients whom did not undergo bronchoscopy. Therefore, it was not possible to characterise them as neutrophil^high^ or neutrophil^low^ (using lung samples) and compare neutrophil response to LPS in these patient groups. We did not use minimum fold change thresholds for gene expression. Instead, we used two cohorts to identify genes of interest, plus protein validation. We assessed protein levels as they are not confounded by macrophage:neutrophil ratio. Some of the small differences in gene expression were associated with greater differences at the protein level, such as TNFA and VEGF, highlighting the value of using different analytical methods to investigate mechanism of interest. The difference in magnitude may be explained by temporal variability between gene and protein expression, or relatively high protein levels due to extra-pulmonary secretion. We limited our analyses to a discrete set of genes to test our hypothesis, but future unbiased investigations in neutrophil^high^ patients would allow a full characterisation of protein levels.

In conclusion, COPD patients with greater distal airway neutrophilic inflammation displayed a gene and protein signature in BAL fluid indicative of increased inflammation and protease activity, increased levels of heme, iron binding and iron containing proteins and evidence of vascular dysfunction. Overall, these results describe a COPD phenotype with excessive neutrophilic inflammation and vascular leakage that could be targeted in future with a precision medicine approach [41].

## Clinical perspectives

- COPD alveolar macrophages and BAL fluid have increased iron levels compared with healthy controls. Vascular leakage has been proposed as a source of increased lung iron in COPD. The association of dysregulated iron metabolism and endothelial dysfunction with increased neutrophilic inflammation in COPD is unknown.
- We show COPD patients with greater distal airway neutrophilic inflammation displayed a signature indicative of increased levels of heme, iron binding and iron containing proteins and evidence of vascular dysfunction.
- Our study describes a COPD phenotype with excessive neutrophilic inflammation and vascular leakage that could be targeted in future with a precision medicine approach.

## Supplementary Material

Supplementary Figures S1-S6 and Tables S1-S4

## Data Availability

The RNA-Seq was uploaded to European genome-phenome archive: EGAD00001002003.

## Open Access

Open access for this article was enabled by the participation of University of Manchester in an all-inclusive Read & Publish agreement with Portland Press and the Biochemical Society under an agreement with JISC.

## Abbreviations

AAT: α-1 antitrypsin
BAL: bronchoalveolar lavage
COPD: chronic obstructive pulmonary disease
CXCL8: C-X-C Motif Chemokine Ligand 8
FC: fold change
FEV_1_: forced expiratory volume in 1 s
FVC: forced vital capacity
HP: haptoglobin
IL6R: interleukin 6 receptor
LCN2: Lipocalin-2
LPS: Lipopolysaccharide
LTF: Lactoferrin
MB: Myoglobin
VEGF: vascular endothelial growth factor
