# Supplementary material for: Dysregulated iron metabolism associates with neutrophilic airway inflammation in COPD
Source: Clin Sci (Lond). 2026 Feb 17;140(3):307–19. doi: 10.1042/CS20257442 (PMC13108851; doi:10.1042/CS20257442)
Supplement: Supplementary Figures S1-S6 and Tables S1-S4 [file CS-2025-7442_supp.pdf]

Supplemental Table 1. Study gene names.

Gene Name	Protein
CXCL1	CXC Motif Chemokine Ligand 1
CXCL2	CXC Motif Chemokine Ligand 2
CXCL3	CXC Motif Chemokine Ligand 3
CXCL5	CXC Motif Chemokine Ligand 5
CXCL6	CXC Motif Chemokine Ligand 6
CXCL7	CXC Motif Chemokine Ligand 7
CXCL8	CXC Motif Chemokine Ligand 8
CXCR2	CXC motif chemokine receptor 2
ALPL	Alkaline phosphatase
IL1A	Interleukin-1 alpha
IL1B	Interleukin-1 beta
TNFA	Tumor necrosis factor alpha
IL6	Interleukin 6
IL6R	Interleukin-6 receptor
MIP1B	Macrophage inflammatory protein-1
IL17A	Interleukin-17A
SAA	Serum amyloid A
NE	Neutrophil elastase
AAT	Alpha-1 antitrypsin
SLPI	Secretory Leukocyte Peptidase Inhibitor
A2M	Alpha-2-Macroglobulin
MMP9	Matrix metalloproteinase 9
TIMP1	TIMP Metallopeptidase Inhibitor 1
A2M	Alpha-2-Macroglobulin
LTF	Lactoferrin
TF	Transferrin
HP	Haptoglobin
CP	Ceruloplasmin
LCN2	Lipocalin-2
HPX	Hemopexin
ABCB6	ATP-binding cassette super-family B member 6
ALAS1	Delta-aminolevulinate synthase 1
ALAS2	Delta-aminolevulinate synthase 2
UROD	Uroporphyrinogen III decarboxylase
UROS	Uroporphyrinogen III Synthase
FECH	Ferrochelatase
PPOX	Protoporphyrinogen oxidase
HMOX1	Heme Oxygenase 1
HMOX2	Heme Oxygenase 2
CEACAM6	Carcinoembryonic antigen-related cell adhesion molecule 6
FTH1	Ferritin heavy chain
FTL	Ferritin light chain
FXN	Frataxin
MB	Myoglobin
TFRC	Transferrin receptor 1
LRP2	Low density lipoprotein receptor-related protein 2
SLC46A1	Proton-coupled folate symporter
SLC11A2	Divalent metal transporter 1
HFE	Hereditary hemochromatosis protein
HFE2	Hemochromatosis type 2
CD163	Cluster of Differentiation 163
LRP1	Low-density lipoprotein receptor-related protein 1
SLC40A1	Solute Carrier Family 40 Member 1
FLVCR1	Feline leukemia virus subgroup C receptor-related protein 1
FLVCR2	Feline leukemia virus subgroup C receptor-related protein 2
ABCG2	ATP-binding cassette super-family G member 2
STEAP3	Metalloreductase STEAP3
SLC25A37	Mitoferrin-1
CYBRD1	Duodenal cytochrome B
ABCB7	ATP-binding cassette sub-family B member 7
ACO1	Aconitase 1
IREB2	Iron Responsive Element Binding Protein 2
HAMP	Hepcidin
HIF1A	Hypoxia Inducible Factor 1 Subunit Alpha
HIF1AN	Hypoxia-inducible factor 1-alpha inhibitor
EPAS1	Endothelial PAS domain-containing protein 1
EGNL1	Hypoxia-inducible factor prolyl hydroxylase 2

ANGPT2	Angiopoietin-2
VEGFA	Vascular endothelial growth factor A
VCAM-1	Vascular cell adhesion molecule 1

Supplemental Table 2. Neutrophilic inflammation marker gene expression and protein levels in neutrophil^{high} and neutrophil^{low} patients. Gene expression was assessed in BAL cells. Protein levels were assessed in BAL fluid. Fold change difference of neutrophil^{high} gene expression and protein levels compared to neutrophil^{low} is also presented. Data is presented as mean ± standard deviations for gene expression and median (range) for BAL fluid protein levels. Comparisons between groups were made by an unpaired t-test for gene expression and protein levels. Red and bold text indicates significant difference between neutrophil^{high} and neutrophil^{low} patients for gene expression in both cohorts and protein levels where data is available and there is a significant difference. CXC Motif Chemokine Ligand 1 (CXCL1), CXC Motif Chemokine Ligand 2 (CXCL2), CXC Motif Chemokine Ligand 3 (CXCL3), CXC Motif Chemokine Ligand 5 (CXCL5), CXC Motif Chemokine Ligand 6 (CXCL7), CXC Motif Chemokine Ligand 7 (CXCL7), CXC Motif Chemokine Ligand 8 (CXCL8), CXC motif chemokine receptor 2 (CXCR2), Alkaline phosphatase (ALPL), Interleukin-1 alpha (IL1A), Interleukin-1 beta (IL1B), Tumor necrosis factor alpha (TNFA), Interleukin 6 (IL6), Interleukin-6 receptor (IL6R), Macrophage inflammatory protein-1 (MIP1B), Interleukin-17A (IL17A), Serum amyloid A (SAA), Neutrophil elastase (NE), Alpha-1 antitrypsin (AAT), Secretory Leukocyte Peptidase Inhibitor (SLPI), Matrix metalloproteinase 9 (MMP9), TIMP Metallopeptidase Inhibitor 1 (TIMP1) and Alpha-2-Macroglobulin (A2M).

**Gene expression**	**EVA**				**Manchester**				**Protein (Manchester)**			
**Chemokines**	**<3%**	**>3%**	**FC**	**P-value**	**<3%**	**>3%**	**FC**	**P-value**	**<3%**	**>3%**	**FC**	**P-value**
CXCL6	**4.0 ± 1.5**	**5.5 ± 2.3**	**1.38**	**0.008**	**1.1 ± 1.2**	**5.0 ± 2.4**	**4.55**	**<0.0001**				
CXCL8	**9.8 ± 1.2**	**13.2 ± 2.5**	**1.35**	**<0.0001**	**10.0 ± 1.6**	**13.1 ± 2.7**	**1.31**	**0.002**	**15.1 (0.8 – 50.5)**	**54.6 (4.7 – 2153)**	**3.62**	**0.002**
CXCL1	**5.5 ± 1.2**	**7.8 ± 2.3**	**1.42**	**0.002**	**6.3 ± 0.6**	**8.5 ± 2.1**	**1.35**	**<0.0001**				
CXCL2	7.6 ± 1.4	8.7 ± 1.6	1.14	0.001	9.7 ± 0.8	10.5 ± 1.5	1.08	0.097				
CXCL3	8.5 ± 1.2	8.9 ± 1.4	1.04	0.2	10.2 ± 0.8	11.0 ± 1.3	1.08	0.07				
CXCL5	11.2 ± 1.4	11.2 ± 1.1	1.00	0.9	10.4 ± 1.1	11.0 ± 1.5	1.06	0.3	2.2 (0.4 – 13.2)	11 (0.5 – 203)	5.00	0.009

CXCL7	7.3 ± 2.4	6.8 ± 2.1	0.93	0.4	7.9 ± 2.0	7.4 ± 1.6	0.94	0.6				
**Inflammation**												
IL17A	**0.4 ± 0.5**	**1.0 ± 1.3**	**2.50**	**0.02**	**0.1 ± 0.5**	**1.7 ± 2.1**	**17.00**	**0.02**				
ALPL	**5.4 ± 1.5**	**8.1 ± 2.0**	**1.40**	**<0.0001**	**4.9 ± 1.5**	**8.8 ± 3.1**	**1.80**	**0.0007**				
SAA	5.4 ± 1.7	6.6 ± 2.1	1.22	0.04	3.6 ± 1.7	5.9 ± 3.4	1.64	0.05				
MIP1B	9.0 ± 0.8	10.6 ± 2.1	1.18	0.0004	8.7 ± 1.1	10.7 ± 2.4	1.22	0.02	3.9 (0.6 – 7.9)	8.1 (0.7 – 280)	2.08	0.1
IL6	4.3 ± 1.4	5.2 ± 1.7	1.21	0.05	4.6 ± 2.0	5.6 ± 2.0	1.22	0.2	0.2 (0.05 – 3.4)	1.1 (0.06 – 59.5)	5.50	0.008
CXCR2	**9.2 ± 0.9**	**10.2 ± 1.4**	**1.10**	**0.004**	**9.7 ± 0.7**	**11.2 ± 1.6**	**1.15**	**0.009**				
IL6R	**9.3 ± 0.8**	**9.9 ± 1.2**	**1.06**	**0.047**	**9.7 ± 0.6**	**11.0 ± 0.8**	**1.13**	**0.0002**	**13.5 (1.1 – 29.9)**	**29.8 (5.2 – 253)**	**2.21**	**0.03**
TNFA	**8.3 ± 0.7**	**8.9 ± 0.9**	**1.07**	**0.01**	**8.6 ± 0.4**	**9.6 ± 1.1**	**1.12**	**0.007**	**0.3 (0.1 – 0.8)**	**1.2 (0.1 – 9.0)**	**4.00**	**0.02**
IL1B	10.5 ± 0.8	11.5 ± 1.6	1.10	0.003	11.2 ± 0.7	12.1 ± 1.6	1.08	0.08	0.4 (0.07 – 2.7)	0.6 (0.04 – 23.7)	1.50	0.5
IL1A	9.4 ± 1.0	9.0 ± 0.9	0.96	0.09	10.0 ± 0.7	9.8 ± 0.7	0.98	0.5	0.01 (0.004 – 0.2)	0.01 (0.005 – 0.7)	1.00	0.9
**Proteases / anti-proteases**												
SLPI	**9.6 ± 1.3**	**11.0 ± 2.2**	**1.15**	**0.006**	**7.3 ± 1.4**	**10.7 ± 2.8**	**1.47**	**0.001**	**8388 (1143 – 89636)**	**42651 (5808 – 715574)**	**5.08**	**0.007**
NE	0.8 ± 1.1	0.7 ± 1.0	0.88	0.5	1.7 ± 1.4	2.3 ± 2.0	1.35	0.4				
MMP9	5.7 ± 1.9	7.6 ± 2.1	1.33	0.001	7.6 ± 1.9	9.2 ± 2.0	1.21	0.06	4654 (149 – 16807)	21576 (1643 – 1123900)	4.63	0.007
TIMP1	11.0 ± 1.0	11.2 ± 1.0	1.02	0.4	12.1 ± 1.0	12.2 ± 1.1	1.01	0.9	468 (20.6 – 4482)	2105 (89.6 – 41284)	4.50	0.02

AAT	16.7 ± 0.8	13.2 ± 0.7	0.79	0.01	17.2 ± 0.5	16.8 ± 0.4	0.98	0.1	98.7 (2.2 – 299)	206 (8.5 – 5953)	2.09	0.03
A2M	11.8 ± 1.2	11.3 ± 1.5	0.96	0.2	13.5 ± 1.3	12.4 ± 1.3	0.92	0.06	160 (6.3 – 330)	400 (64.9 – 4510)	2.50	0.01

Supplemental table 3. Iron metabolism gene expression and protein levels in neutrophil^{high} and neutrophil^{low} patients. Gene expression was assessed in BAL cell. Protein levels were assessed in BAL fluid. Fold change difference of neutrophil^{high} gene expression and protein levels compared to neutrophil^{low} is also presented. Data is presented as mean ± standard deviations for gene expression. Data is presented as median (range) for BAL fluid protein levels. Comparisons between groups were made by an unpaired t-test for gene expression and protein levels. Red text indicates significant difference between neutrophil^{high} and neutrophil^{low} patients for gene expression in both cohorts and protein levels where data is available and there is a significant difference. Lactoferrin (LTF), Haptoglobin (HP), Ceruloplasmin (CP), Lipocalin-2 (LCN2), Delta-aminolevulinate synthase 1 (ALAS1), Heme Oxygenase 2 (HMOX2), Carcinoembryonic antigen-related cell adhesion molecule 6 (CEACAM6), Ferritin heavy chain (FTH1 Frataxin (FXN), Myoglobin (MB), Mitoferrin-1 (SLC25A37), Vascular cell adhesion protein 1 (VCAM-1), Vascular endothelial growth factor A (VEGFA) and Angiopoietin-2 (ANGPT2).

**Gene expression**	**EVA**				**Manchester**				**Manchester (Protein)**			
**Iron binding**	**<3%**	**>3%**	**FC**	**P-value**	**<3%**	**>3%**	**FC**	**P-value**	**<3%**	**>3%**	**FC**	**P-value**
LTF	**4.2 ± 1.4**	**5.8 ± 2.0**	**1.4**	**0.002**	**2.7 ± 2.0**	**5.8 ± 3.1**	**2.15**	**0.01**	**10.5 (0.5 – 73)**	**49 (2.4 – 204)**	**4.67**	**0.01**
CP	**6.2 ± 1.5**	**7.6 ± 2.6**	**1.22**	**0.02**	**4.3 ± 1.1**	**7.7 ± 2.3**	**1.79**	**0.0001**				
LCN2	**7.7 ± 1.5**	**9.3 ± 2.4**	**1.21**	**0.004**	**5.8 ± 1.9**	**9.2 ± 3.2**	**1.58**	**0.005**				
HP	11.8 ± 3.0	10.9 ± 2.4	0.92	0.3	11.1 ± 3.1	10.5 ± 3.8	0.95	0.7	0.02 x10^{-6} (1.5 x10^{-9} – 5.3 x10^{-8})	2.3 x10^{-6} (7.5 x10^{-9} – 3.6 x10^{-5})	115	0.01
**Iron import**												
B2M	18.6 ± 0.7	18.2 ± 0.6	0.98	0.04	18.0 ± 0.6	18.1 ± 0.4	1.00	0.7	0.01 (0.0004 – 0.03)	0.05 (0.003 – 0.4)	5	0.04
**Heme synthesis**												
ALAS1	**13.0 ± 0.8**	**12.3 ± 0.7**	**0.95**	**0.002**	**13.6 ± 0.6**	**13.0 ± 0.6**	**0.96**	**0.03**				
**Heme degradation**												
CEACAM6	**6.9 ± 1.3**	**8.2 ± 2.1**	**1.18**	**0.01**	**5.4 ± 1.8**	**8.7 ± 1.9**	**1.61**	**0.0004**				
HMOX2	**10.8 ± 0.7**	**10.3 ± 0.6**	**0.95**	**0.009**	**11.6 ± 0.5**	**11.2 ± 0.3**	**0.97**	**0.02**				
**Iron storage**												
MB	**3.9 ± 1.5**	**5.1 ± 2.1**	**1.3**	**0.02**	**1.8 ± 1.6**	**4.8 ± 2.8**	**2.67**	**0.004**	**0.4 (0.017 – 0.98)**	**1.5 (0.092 – 4.7)**	**3.75**	**0.01**
FTH1	18.32 ± 0.7	18.0 ± 0.6	0.98	0.19	15.0 ± 0.3	14.7 ± 0.6	0.98	0.2	99 (8.5 – 421)	50 (1.5 – 144)	0.51	0.2
FXN	**7.9 ± 0.7**	**7.3 ± 0.6**	**0.92**	**0.004**	**8.6 ± 0.3**	**8.3 ± 0.2**	**0.97**	**0.009**				
**Intracellular iron trafficking**												
SLC25A37	**7.8 ± 0.9**	**9.1 ± 1.4**	**1.17**	**<0.0001**	**8.7 ± 0.7**	**10.9 ± 2.0**	**1.25**	**0.002**				
**Vascular remodelling**												
VCAM-1	4.7 ± 1.7	4.7 ± 1.7	1.00	0.99	2.2 ± 2.5	3.8 ± 2.2	1.73	0.1	0.04 (0.002 – 0.1)	0.3 (0.007 – 1.9)	7.5	0.05
VEGFA	**7.5 ± 1.2**	**8.4 ± 1.7**	**1.12**	**0.02**	**8.6 ± 1.0**	**10.4 ± 1.6**	**1.21**	**0.003**	**16 (0.53 – 41)**	**55 (2.5 – 220)**	**3.43**	**0.02**

Fibrinogen			0.4×10^{-4} (6.2×10^{-7} – 1.0×10^{-4})	3.2×10^{-4} (3.2×10^{-4} – 2.5×10^{-3})	8	0.02
ANGPT2	7.3 ± 1.0 6.3 ± 1.2 0.86 0.001	7.8 ± 0.7 7.6 ± 1.0 0.97 0.3	4×10^{-3} (1×10^{-3} – 0.01)	0.02 (0.001 – 0.08)	50	0.0002

Supplemental Table 4. Additional iron metabolism gene expression and protein levels in neutrophil^{high} and neutrophil^{low} patients. Gene expression was assessed in BAL cells. Protein levels were assessed in BAL fluid. Fold change difference of neutrophil^{high} gene expression and protein levels compared to neutrophil^{low} is also presented. Data is presented as mean ± standard deviations for gene expression and median (range) for BAL fluid protein levels. Comparisons between groups were made by an unpaired t-test for gene expression and protein levels. Transferrin (TF), Hemopexin (HPX), ATP-binding cassette super-family B member 6 (ABCB6), Delta-aminolevulinate synthase 2 (ALAS2), Uroporphyrinogen III decarboxylase (UROD), Uroporphyrinogen III Synthase (UROS), Ferrochelatase (FECH), Protoporphyrinogen oxidase (PPOX), Heme Oxygenase 1 (HMOX1),), Ferritin light chain (FTL), Transferrin receptor 1 (TFRC), Low density lipoprotein receptor-related protein 2 (LRP2), Proton-coupled folate symporter (SLC46A1), Divalent metal transporter 1 (SLC11A2), Hereditary hemochromatosis protein (HFE), Hemochromatosis type 2 (HFE2), Cluster of Differentiation 163 (CD163), Low-density lipoprotein receptor-related protein 1 (LRP1), Solute Carrier Family 40 Member 1 (SLC40A1), Feline leukemia virus subgroup C receptor-related protein 1 (FLVCR1), Feline leukemia virus subgroup C receptor-related protein 2 (FLVCR2), ATP-binding cassette super-family G member 2 (ABCG2), Metalloreductase (STEAP3), Duodenal cytochrome B (CYBRD1), ATP-binding cassette sub-family B member 7 (ABCB7), Aconitase 1 (ACO1), Iron Responsive Element Binding Protein 2 (IREB2), Hepcidin (HAMP), Hypoxia Inducible Factor 1 Subunit Alpha (HIF1A), Hypoxia-inducible factor 1-alpha inhibitor (HIF1AN), Endothelial PAS domain-containing protein 1, EGNL1 (EPAS1) and Hypoxia-inducible factor prolyl hydroxylase 2 (EPAS1).

**Gene expression**	**EVA**				**Manchester**			
**Iron binding**	**<3%**	**>3%**	**FC**	**P-value**	**<3%**	**>3%**	**FC**	**P-value**
TF	1.3 ± 1.4	2.6 ± 1.9	2.0	0.007	3.1 ± 1.1	3.2 ± 1.8	1.03	0.9
**Heme synthesis**								
ALAS2	3.26 ± 2.3	4.4 ± 2.6	1.35	0.1	1.4 ± 2.0	4.2 ± 2.8	3.0	0.01
UROD	10.6 ± 0.7	10.2 ± 0.6	0.96	0.01	11.5 ± 0.7	11.1 ± 0.5	0.97	0.07
UROS	8.9 ± 0.8	8.4 ± 0.5	0.94	0.1	10.0 ± 0.6	9.5 ± 0.4	0.95	0.02
FECH	10.0 ± 0.8	9.4 ± 0.6	0.94	0.003	10.5 ± 0.4	10.5 ± 0.4	1.0	0.9
**Heme degradation**								
HMOX2	10.8 ± 0.7	10.3 ± 0.6	0.95	0.009	11.6 ± 0.5	11.2 ± 0.3	0.97	0.02
CEACAM6	6.9 ± 1.3	8.2 ± 2.1	1.18	0.01	5.4 ± 1.8	8.7 ± 1.9	1.61	0.0004
**Iron import**								
TFRC	15.6 ± 0.9	15.0 ± 0.9	0.96	0.02	15.4 ± 0.7	15.9 ± 0.7	1.03	0.07
TFR2	3.6 ± 1.0	3.1 ± 1.2	0.86	0.09	4.8 ± 0.5	5.3 ± 0.5	1.10	0.04
HFE	9.6 ± 0.8	9.0 ± 0.73	0.94	0.006	10.0 ± 0.7	9.8 ± 0.6	0.98	0.5
CD163	15.0 ± 0.9	14.4 ± 0.7	0.96	0.02	15.2 ± 0.4	15.2 ± 0.5	1.0	0.9
**Iron export**								
SLC40A1	7.8 ± 1.2	8.1 ± 1.4	1.04	0.5	6.2 ± 1.2	8.2 ± 1.2	1.32	0.0006
FLVCR2	12.3 ± 0.8	11.6 ± 0.7	0.94	0.002	12.6 ± 0.3	12.4 ± 0.4	0.98	0.2
ABCG2	10.8 ± 0.8	10.0 ± 0.8	0.93	0.003	10.9 ± 0.5	10.9 ± 1.0	1.0	0.9
**Intracellular iron trafficking**								
STEAP3	10.9 ± 0.8	10.4 ± 0.7	0.95	0.01	11.2 ± 0.3	11.1 ± 0.4	0.99	0.7
ABCB7	9.3 ± 0.7	8.7 ± 0.6	0.94	0.003	9.9 ± 0.3	9.7 ± 0.3	0.98	0.2
**Transcription factors and hormones**								
ACO1	13.0 ± 0.7	12.2 ± 0.9	0.94	0.004	12.8 ± 0.4	12.8 ± 0.5	1.0	0.9
IREB2	11.0 ± 0.8	10.5 0.7	0.95	0.01	11.2 ± 1.0	11.7 ± 0.6	1.04	0.16
**Hypoxia inducible factors**								

HIF1A	11.1 ± 1.0	11.4 ± 1.0	1.03	0.4	10.8 ± 1.2	12.1 ± 0.8	1.12	0.006
HIF1AN	10.6 ± 0.8	10.2 ± 0.6	0.96	0.06	10.4 ± 0.3	10.7 ± 0.2	1.02	0.01
EPAS1	12.6 ± 0.9	12.1 ± 0.7	0.96	0.01	12.8 ± 0.5	13.0 ± 0.5	1.01	0.2
EGLN1	10.7 ± 0.8	10.2 ± 0.6	0.95	0.02	10.6 ± 0.5	11.0 ± 0.2	1.03	0.05

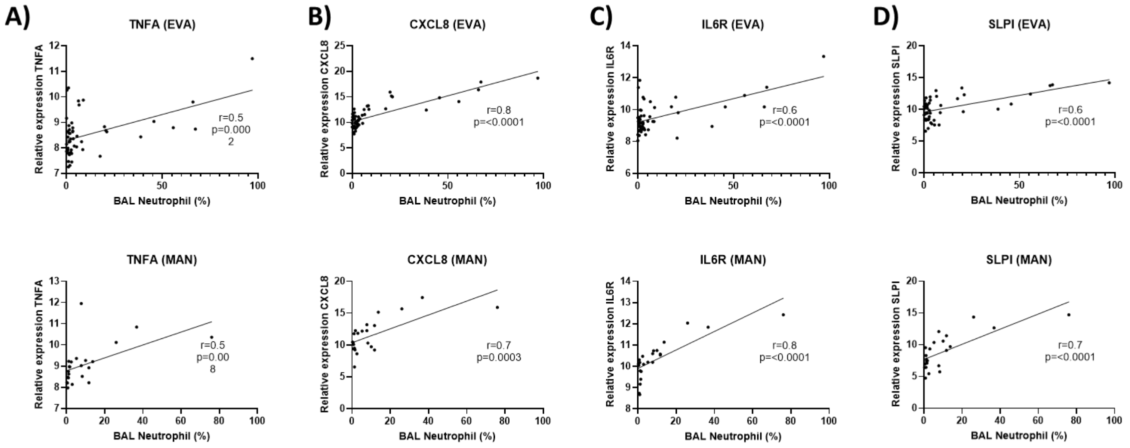

Supplemental Figure 1. BAL cell neutrophil gene expression in neutrophill^{ow} and neutrophil^{high} chronic obstructive pulmonary disease (COPD) patients correlation with BAL cell neutrophil percentage. RNA-sequencing was used to examine the expression A) TNFA, B) CXCL8 C) IL6R and D) SPLI in Eva: neutrophil^{low} n=22 and neutrophil^{high} n=29 and Manchester: neutrophil^{low} n=11 and n=12 neutrophil^{high} COPD patients. Data are presented as individual values with linear regression plotted.

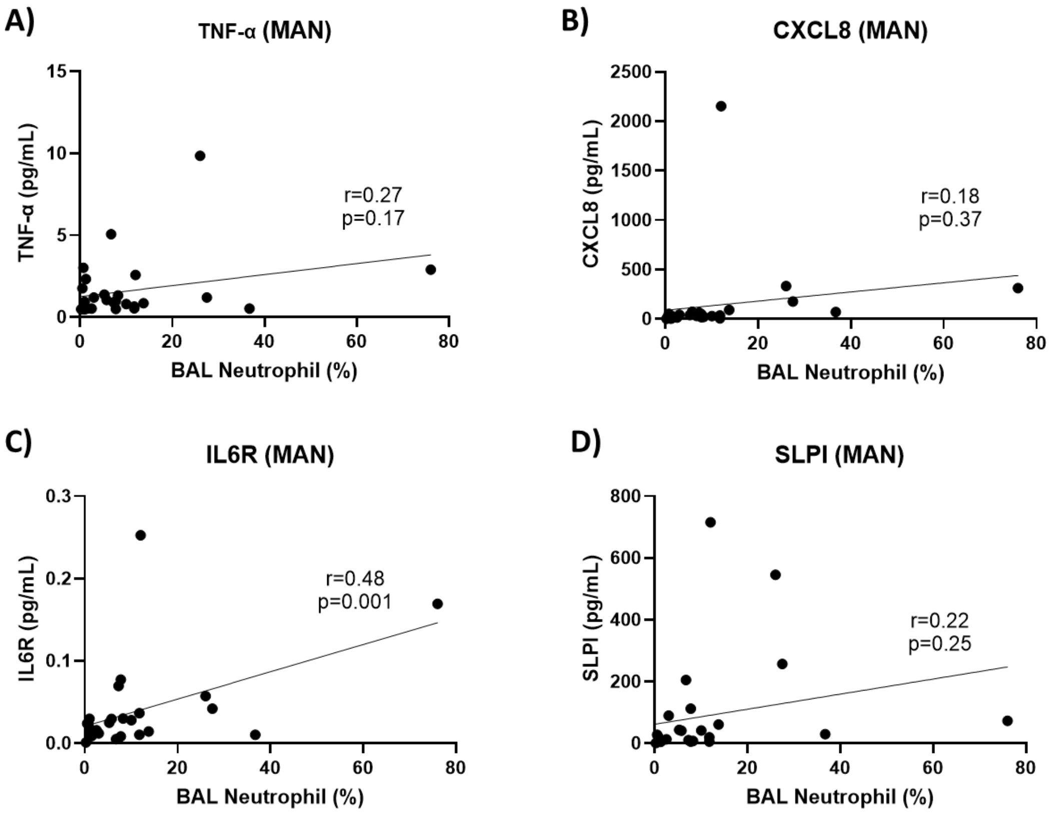

Supplemental Figure 2. BAL cell neutrophil protein levels in neutrophil^{low} and neutrophil^{high} chronic obstructive pulmonary disease (COPD) patients correlation with BAL cell neutrophil percentage. Protein levels were detected by a multiplex assay (myriad RBM) was used to examine the levels of A) TNFA, B) CXCL8 C) IL6R and D) SPLI in the Manchester cohort: neutrophil^{low} n=11 and n=12 neutrophil^{high} COPD patients. Data are presented as individual values with linear regression plotted.

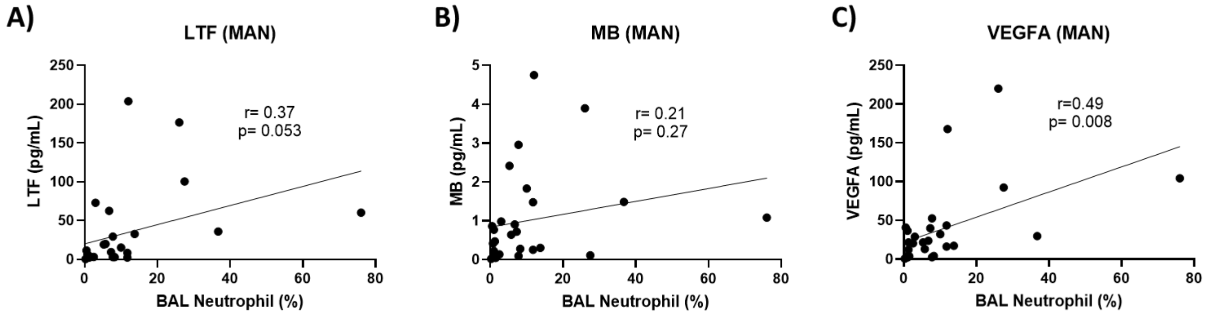

Supplemental Figure 3. BAL iron and endothelial protein levels in neutrophil^{low} and neutrophil^{high} chronic obstructive pulmonary disease (COPD) patients correlation with BAL cell neutrophil percentage. Protein levels were detected by a multiplex assay (myriad RBM) was used to examine the levels of A) LTF, B) MB and C) VEGFA in the Manchester cohort: neutrophil^{low} n=11 and n=12 neutrophil^{high} COPD patients. Data are presented as individual values with linear regression plotted.

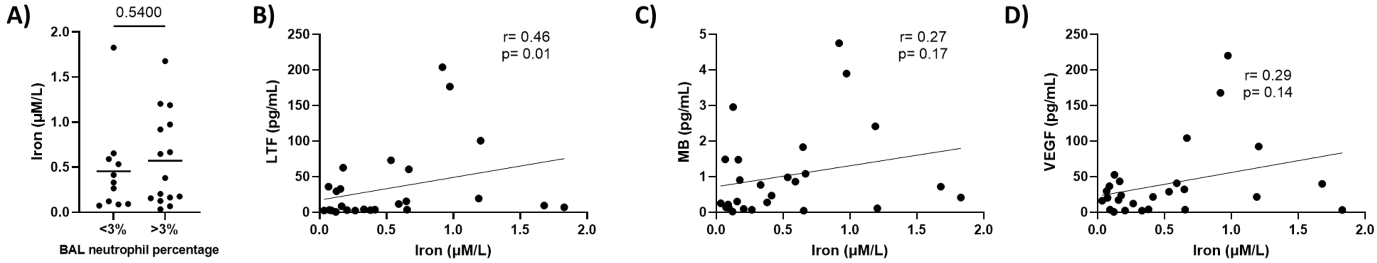

Supplemental Figure 4. BAL iron and iron/endothelial protein levels in neutrophil^{low} and neutrophil^{high} chronic obstructive pulmonary disease (COPD) patients. A) BAL iron was measured by Inductively coupled plasma mass spectrometry (ICP-MS) in the Manchester cohort: neutrophil^{low} n=11 and n=15 neutrophil^{high} COPD patients. Data are presented as individual values where the black horizontal line presents the mean. BAL iron was plotted against B) LTF, C) MB and D) VEGF protein levels. Data are presented as individual values with linear regression plotted.

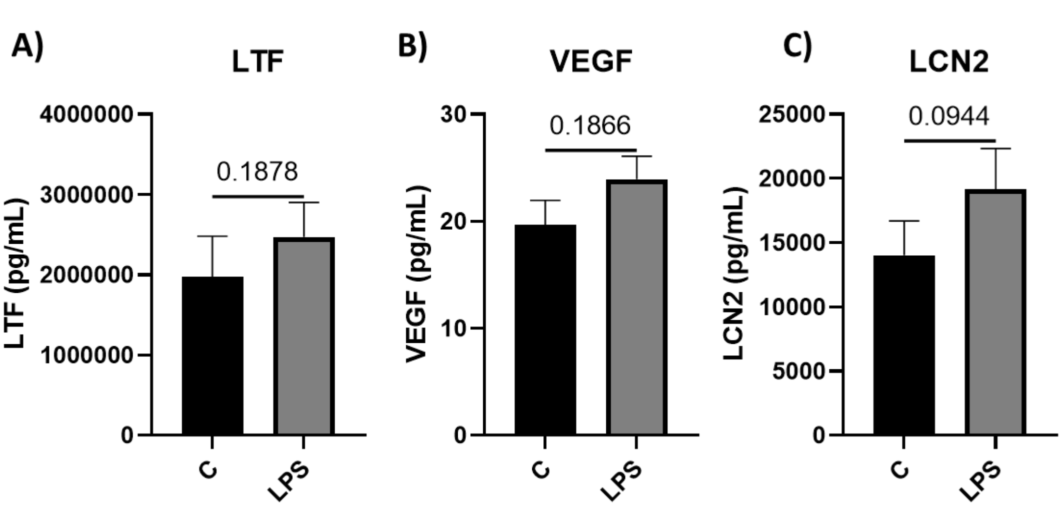

Supplemental Figure 5. Iron and endothelial marker expression in activated neutrophils. Blood neutrophils isolated from COPD patients (n=8) were treated with 0.1 ug/mL of LPS for 3 hours. Protein secretion of A) LTF, B) VEGFA and C) LCN2 was measured by ELISA. Comparisons between groups were made by a paired t-test.

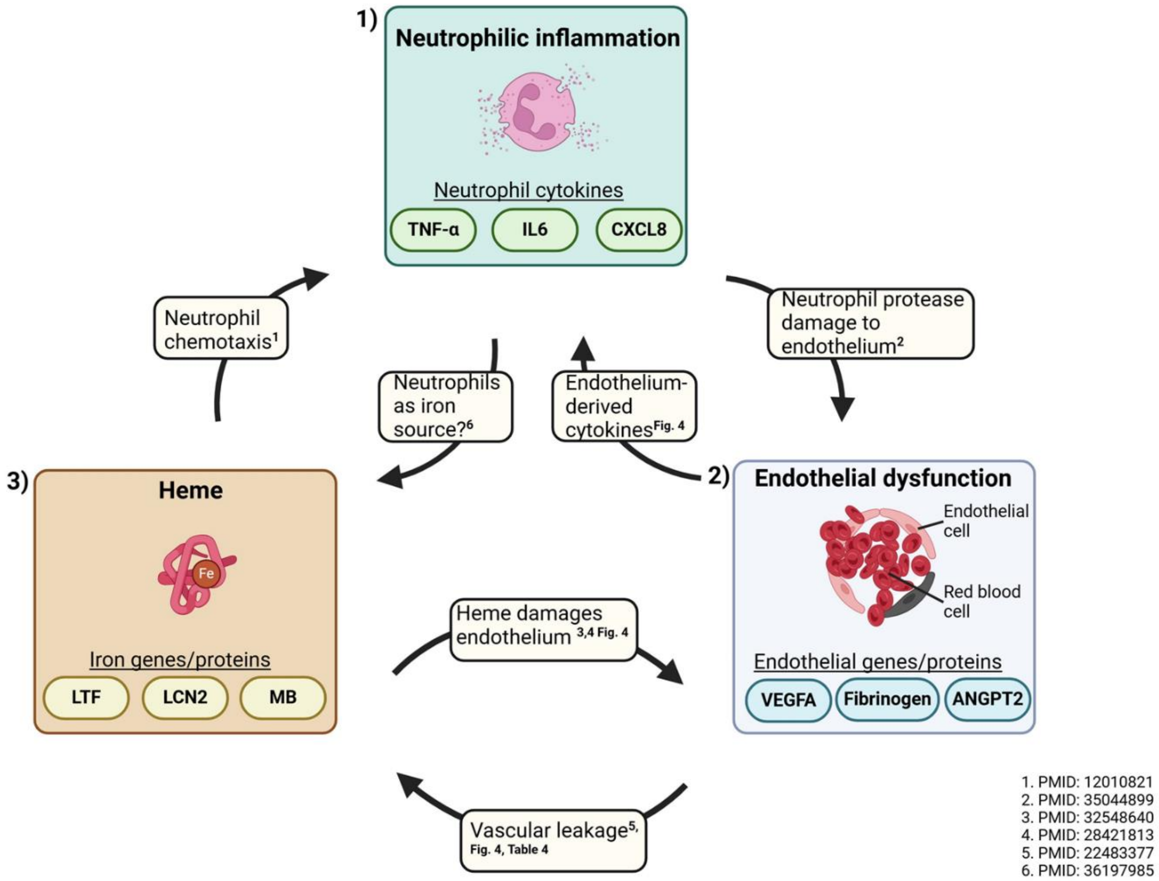

Supplemental Figure 6. Schematic diagram of proposed mechanism of the associations of neutrophilic inflammation, endothelial dysfunction and heme in COPD.
